# One aptamer, two functions: the full-length aptamer inhibits AMPA receptors, while the short one inhibits both AMPA and kainate receptors

**Published:** 2017-06-12

**Authors:** William J. Jaremko, Zhen Huang, Wei Wen, Andrew Wu, Nicholas Karl, Li Niu

**Affiliations:** Department of Chemistry and Center for Neuroscience Research, University at Albany, SUNY, Albany, New York 12222, USA

**Keywords:** AMPA receptors, kainate receptors, SELEX, RNA aptamers

## Abstract

AMPA and kainate receptors, along with NMDA receptors, are distinct subtypes of glutamate ion channels. Excessive activity of AMPA and kainate receptors has been implicated in neurological diseases, such as epilepsy and neuropathic pain. Antagonists that block their activities are therefore potential drug candidates. In a recent article in the *Journal of Biological Chemistry* by Jaremko *et al*. 2017, we have reported on the discovery and molecular characterization of an RNA aptamer of a dual functionality: the full-length RNA (101 nucleotide) inhibits AMPA receptors while the truncated or the short (55 nucleotide) RNA inhibits both the AMPA and kainate receptors. The full-length RNA aptamer was isolated through a specially designed, systematic evolution of ligands by exponential enrichment (SELEX) using only a single type of AMPA receptors expressed in HEK-293 cells. The design feature and the results of our recent article are highlighted here, as they demonstrate the utility of the SELEX approach and the potential of using a single AMPA receptor type to develop potent, novel RNA aptamers targeting multiple subunits and AMPA/kainate receptor subtypes with length-dependent functionalities.

Developing RNA therapeutics for treatment of a disease could begin from using SELEX ^[1–3]^ to screen and isolate candidate RNAs from a large RNA library against the target of interest. Such an exercise normally involves a single target (e.g., protein or receptor) with the intent of identifying aptamers with a single target specificity. Indeed, aptamers that can even discriminate against different conformations ^[4]^, different functional groups ^[5, 6]^, and even an amino acid mutation ^[7]^ on a target have been reported. To achieve target specificity through SELEX, one routinely uses a strong chemical pressure, such as an inhibitor that binds to the same site or mutually exclusive sites on that single target, in order to displace and enrich target-specific aptamers through *in vitro* evolution. However, there could be a utility of using the same target in SELEX to purposely evolve RNA aptamers with intended actions against multiple targets for drug discovery. This approach is based on the assumption that these targets are all involved in a disease. This approach may be especially useful with the application of SELEX to isolating aptamers against membrane proteins that must be expressed in a heterologous expression system such as HEK-293 cells. Expressing a single target, i.e., one protein or receptor, to maximize the surface expression and density as opposed to expressing multiple receptors with “diluted” surface density for any one of the targets would be an advantage for this approach. In a recent article in the *Journal of Biological Chemistry* by Jaremko *et al*. 2017^[8]^, we have reported such an approach for the discovery and molecular characterization of an RNA aptamer that has a length-dependent, dual functionality against AMPA and kainate receptors.

AMPA and kainate receptors, along with the *N*-methyl-D-aspartate (NMDA) receptors, are distinct subtypes of the glutamate ion channel receptor family. These receptors mediate the majority of excitatory neurotransmission in the mammalian central nervous system (CNS) and are indispensable for brain development and function ^[9–11]^. AMPA and kainate receptors are more alike in both sequence and structure, as compared with NMDA receptors ^[11, 12]^. AMPA receptors have 4 subunits, GluA1–4, while kainate receptors have five subunits, GluK1–5. Functionally, AMPA receptors are expressed post-synaptically and are involved in fast excitatory neurotransmission ^[9]^. Kainate receptors are expressed both pre- and post-synaptically; they contribute to excitatory neurotransmission and also modulate network excitability by regulating neurotransmitter release ^[13, 14]^.

AMPA and kainate receptors are involved in neurological diseases. A study of GluK2-deficient mice has revealed that hippocampal neurons in the CA3 region express both AMPA and kainate receptors, and both receptor types are involved in seizures ^[15]^. Entorhinal cortex, a highly epilepsy-prone brain region, also expresses GluA1–4 and GluK5 ^[16]^. In both human patients and animal models of temporal lobe epilepsy, the axons of granule cells that normally contact CA3 pyramidal cells sprout to form aberrant glutamatergic excitatory synapses onto dentate granule cells ^[17–19]^. The formation of aberrant mossy fiber synapses onto dentate granule cells has been suggested to induce the recruitment of kainate receptors in chronic epileptic rats. These granule cells express AMPA receptors as well, especially GluA1 and GluA2 subunits ^[20]^. Another example of a neurological disorder that involve both AMPA and kainate receptor types is acute and chronic pain mediated through interior cingulate cortex ^[21, 22]^. Interestingly, AMPA and KA receptors have been also implicated in osteoarthritis and rheumatoid arthritis ^[23]^. Specifically, both receptors are expressed in human arthritis tissue. In rat models of antigen-induced arthritis, the use of the AMPA/KA receptor antagonist NBQX was shown to alleviate inflammation, pain and joint degeneration ^[23]^. These lines of evidence therefore suggest that antagonists capable of blocking the activity of both AMPA and kainate receptors *in vivo* should be therapeutically useful. In fact, a nonselective AMPA/kainate receptor inhibitor, tezampanel (NGX424; Torrey Pines Pharmaceutics), reduced both migraine pain and other symptoms in a Phase II trial. NS1209 (NeuroSearch A/S), another nonselective AMPA/kainate receptor antagonist, was also shown in Phase II studies to alleviate refractory status epilepticus and neuropathic pain ^[24]^. In this context, RNA aptamers with dual actions on both AMPA and kainate receptors would be a class of water-soluble antagonists, alternative to small-molecule inhibitors.

The hypothesis we tested was based on the assumption that an RNA exerts a variety of tertiary interactions with its target(s) (i.e., hydrophobic and electrostatic interactions, hydrogen bonding and van der Walls forces) ^[25]^, and the types and the strengths of these interactions should be length (and sequence) dependent. If we can find an aptamer that covers a sufficient range of these interactions with two targets, it is possible that different subsets of these interactions may be differentially used for the two targets – truncation of the length, thereby fine-tuning these subsets of interactions, may decouple the differential molecular recognitions and specificities. To test this hypothesis, namely finding an aptamer that may act on both AMPA and kainate receptors but by using a single receptor as the target of selection in SELEX, we designed our approach based on the following rationale. (i) AMPA and kainate receptors share a high degree of sequence and structural homologies ^[10, 12]^. (ii) Given its size (100 nucleotides in length as in our library), an RNA may bind to the surface of a receptor topologically. As a result, the larger area of interaction with the receptor, as compared with the interaction of a small molecule, may generate a range of size-dependent, multivalent binding interactions so that an RNA could bind to and inhibit AMPA and kainate receptors. In contrast, using multiple targets may likely lead to the identification of individual aptamers with singular activity. (iii) We further decided to choose an AMPA receptor, rather than a kainate receptor, as that single receptor target for SELEX, based on the fact that there are far more inhibitors of AMPA receptors ^[26]^ than those of kainate receptors ^[27]^. Developing antagonists against kainate receptors in general has been far more challenging ^[27]^. Among all possible AMPA receptor types, we chose GluA1/2R as the target of selection. GluA1/2R is an important channel type found *in vivo* ^[28–31]^; here GluA2R is the edited isoform at the glutamine/arginine (Q/R) site of the GluA2 AMPA receptor subunit. (iv) Finally, instead of choosing an inhibitor or a binder to displace RNAs, we used urea after the binding step to denature both the target and possibly all bound RNA molecules, thereby allowing us to select and screen the maximal number of RNA molecules bound to all possible sites on the target. It should be further noted that the choice of using GluA1/2R AMPA receptor channel type seemed more appropriate, because to date, there is no known selective antagonist targeting the GluA1/2R channel type. Therefore, all of these design features were intended to maximize the possibility of finding an RNA aptamer potentially with a broad range of activity.

Using our design approach and SELEX, we successfully isolated an aptamer, which we termed as “AB9” aptamer (101 nt), and a series of truncations of the full-length RNA led to a 55-nt RNA, which we termed as “AB9s” (Figure 1) ^[8]^. There are several interesting features about AB9 and its short version AB9s. First, the full-length aptamer inhibits AMPA receptors (red columns symbolize all AMPA receptor subunits in Figure 1). In fact, AB9 is more selective towards the GluA1/2R, the SELEX target. Not surprisingly, AB9 also inhibits GluA1 and 2 AMPA receptor subunits. However, AB9s, the short, 55-nt RNA, inhibits both the AMPA and kainate receptors (Figure 1, the lower bar graph, purple columns for the kainate receptors). Second, AB9 and AB9s appear to have different binding profiles and dissociation kinetics, measured with the use of ^32^P-labeled aptamers. AB9 is slower to dissociate from the target, whereas AB9s is fast, suggesting a difference in interacting with their respective sites. Removing the central sequence segment (see secondary structures as predicted by MFold in Figure 1) has turned AB9 into a much better inhibitor of the GluK1 and GluK2, the two key kainate receptor subunits, although the origin of this enhancement due to a smaller size, as compared with the full size aptamer, is unclear at the present. Nonetheless, these results are consistent with the assumption that both receptors share high degree structural homology and perhaps even a high degree of homology in sites or “druggable” sites. As expected, neither aptamer has any effect on NMDA receptors (Figure 1, bar graphs).

Our aptamers are better than the existing AMPA and kainate receptor antagonists by several measures. First, virtually all known kainate receptor antagonists generally have a stronger selectivity towards GluK1 than any other kainate receptor subunits ^[27]^. Almost all that inhibit GluK2 actually have stronger potency towards GluK1 ^[27]^. In this context, it is unique that AB9s is nearly equally effective in inhibiting both the GluK1 and GluK2 kainate receptors. Second, AB9s further possesses a nearly identical potency for both the AMPA and kainite receptors (Figure 1). Yet, among the existing inhibitors of either AMPA or kainate receptors, NBQX does inhibit roughly equally well both GluK1 and GluK2; but NBQX is considered an AMPA receptor inhibitor, because it inhibits AMPA receptors >8-fold stronger than it does on kainate receptors ^[27]^. Glutamylaminomethyl sulfonic acid marginally distinguishes kainate from AMPA receptors, based on various *in vivo* and *in vitro* tests, including a test in a seizure model ^[32–35]^. Yet, GAMS shows a significant antagonism on NMDA receptors ^[36]^. In contrast, AB9s can block the activity of both AMPA and kainate receptors equally well without appreciable NMDA receptor activity. In addition, because the aptamer is an RNA molecule, it is a water soluble antagonist, different from almost all of the existing antagonists for either AMPA or kainate receptors.

The experimental design by which we used a single SELEX target (i.e., GluA1/2R) in a single SELEX operation to evolve a single RNA aptamer that acts on both the AMPA and kainate receptors, depending on its length, turns out to be an effective way of generating RNA inhibitors with a desirable inhibitory versatility. It should be noted that the success of this approach relies on high degree sequence and structural similarities not only between the kainate and AMPA receptor subtypes but also within a single receptor subtype. More precisely, no place shows a higher structural similarity than the site to which AB9 binds, although at the moment, we do not know where this site is. We do know, however, this site is a noncompetitive one ^[8]^. It is highly likely that the “footprint” of AB9 site covers a larger surface area, which is needed to inhibit more selectively AMPA receptors. A short version (AB9s), however, uses perhaps only partial footprint, enough for recognizing and effectively inhibiting kainate receptors. In fact, as seen in the two bar graphs, the enhancement of the kainate receptor antagonism in the short RNA aptamer is actually at the expense of diminishing slightly the AMPA receptor potency. Finally, the existence of this site(s), full or partial, further suggests a possibility of developing chemically modified RNA aptamers amenable for *in vivo* application as therapeutic RNAs.

## Figures and Tables

**Figure 1:**
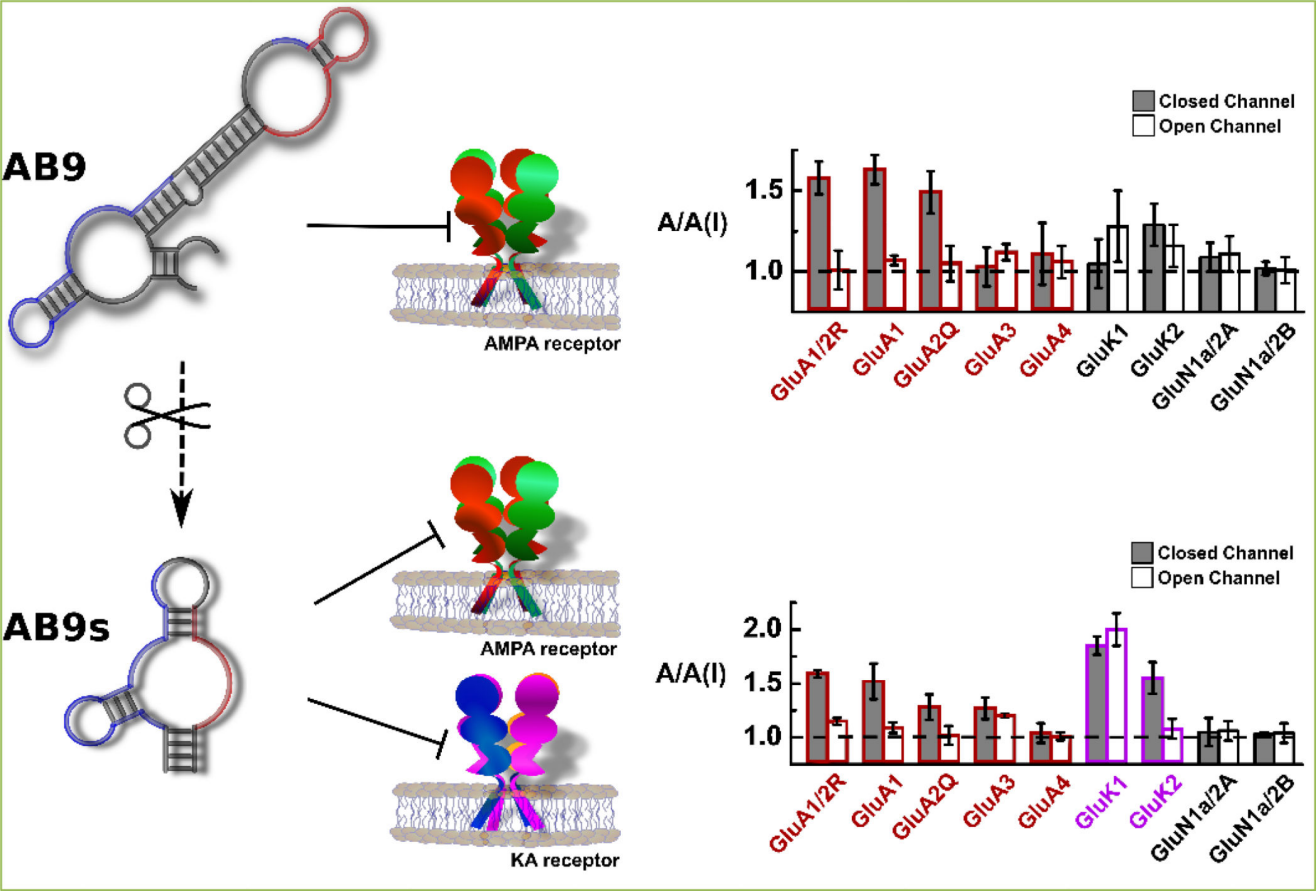
AB9 and AB9s inhibit AMPA and kainate receptors in a length dependent manner The Mfold predicted structures for AB9 and AB9s are shown on the left. The red and blue regions represent sequence stretches essential for function (inhibition). AB9 (full length, 101 nucleotides in length) inhibits more selectively AMPA receptors, whereas AB9s (55 nucleotides in length inhibits both AMPA and KA receptors (see the bar graphs on the right). In the middle are two cartoon drawings of the AMPA receptors (red/green) and kainate receptors (blue/purple). For functional assay, each receptor was transiently expressed in HEK-293 cells. Whole-cell current recording was used to measure the whole-cell current amplitude in the absence, A, and presence of an aptamer, A(I) (in these bar graphs, 2 µM aptamer was used in each assay). The potency and the selectivity of an aptamer against the open-channel, and the closed-channel state are assayed ^[8]^.

## Abbreviations

AMPA: α-amino-3-hydroxy-5-methyl-4-isoxazolepropionic acid
CNS: central nervous system
HEK-293 cells: human embryonic kidney cells
NMDA: *N*-methyl-D-aspartate
nt: nucleotide
SELEX: systematic evolution of ligands by exponential enrichment.
